# Commentary: Identification of cuproptosis hub genes contributing to the immune microenvironment in ulcerative colitis using bioinformatic analysis and experimental verification

**DOI:** 10.3389/fimmu.2023.1224127

**Published:** 2023-08-18

**Authors:** Shaopeng Sun, Bin Lv

**Affiliations:** Department of Gastroenterology, The First Affiliated Hospital of Zhejiang Chinese Medical University (Zhejiang Provincial Hospital of Chinese Medicine), Hangzhou, China

**Keywords:** inflammatory bowel disease, ulcerative colitis, cuproptosis, bioinformatics, FDX1

Cuproptosis is an entirely novel copper-dependent regulated cell death that differs morphologically, biochemically, and genetically from apoptosis, autophagy and ferroptosis (1). Yang et al. (2) and Chen et al. (3) conducted studies that suggested a potential therapeutic role of cuproptosis in inflammatory bowel disease (IBD). They reached this conclusion by employing bioinformatics analysis and experimental validation using dextran sodium sulfate (DSS)-induced mouse models. Yang et al. confirmed the therapeutic effects of three cuproptosis-related genes (*DLAT, DLD*, and *PDHA1*) on immune infiltration in patients with ulcerative colitis (UC). Similarly, Chen et al. found that Crohn’s disease (CD), UC, celiac disease (CEL), and IBD-induced cancer (IBD-CA) had common cuproptosis-related differentially expressed genes, including *DLAT, LIAS, DBT*, and *PDHA1*. Both these studies had positive implications for mechanism exploration; however, further analyses of their results are warranted.

Tsvetkov P et al. first discovered cuproptosis in 2022 (1), and since then, 12 cuproptosis-related genes, namely *FDX1, LIAS, DLAT, DLD, PDHA1, PDHB, MTF1, GLS, CDKN2A, LIPT1, ATP7B*, and *SLC31A1*, have been validated. This gene set is currently used in many disease studies using bioinformatics analysis. Especially, *DLAT, DLA, PDHA1, LIAS*, and *DBT* are known to promote cuproptosis (**Figure 1**); hence, the downregulation of these genes prevents cuproptosis. Of note, the promotion of tumor cell death and inhibition of non-tumor cell death are opposing treatment strategies. For example, ferroptosis inhibition alleviates experimental colitis (4) while ferroptosis promotion in tumor cells is a novel therapeutic approach for cancer (5). However, in the above-mentioned studies, the expression of these genes in the intestinal mucosa samples of IBD was found to be downregulated compared to individuals without IBD (**Figure 1**). According to Tsvetkov P et al. (1), *FDX1* is a key cuproptosis regulator that reduces Cu^2+^ to the more toxic Cu^+^, thus promoting abnormal oligomerization of thioacylated proteins in the tricarboxylic acid cycle. However, Yang et al. (2) found *FDX1* expression to be downregulated in patients with IBD compared to normal individuals. This seems contradictory if we assume that cuproptosis occurs in the intestinal mucosa of patients with IBD. The contradictory results suggest two things: (I) the downregulation of these genes may not be related to cuproptosis, and may only be as a result of the disease itself; (II) The regulatory mechanism of cuproptosis in normal intestinal mucosal cells could be opposite to that in tumor cells. These hypotheses require further validation.

**Figure 1:**
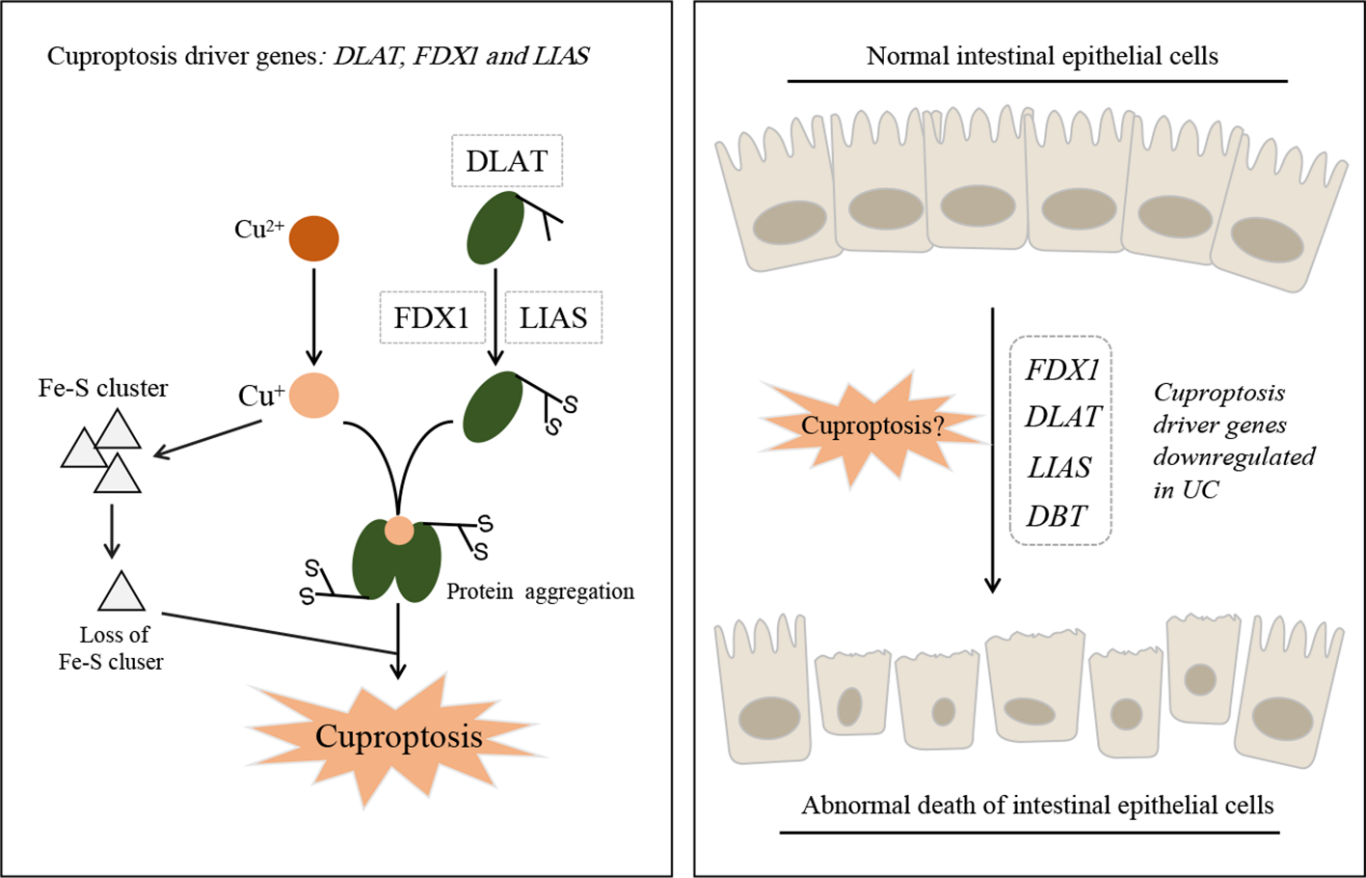
Schematic model of cuproptosis and ulcerative colitis. Cu^+^ binds to lipoylated mitochondrial enzymes DLAT and induced the aggregation of these proteins. Mitochondrial ferredoxin (FDX1) and lipoyl synthase (LIAS) are key driver genes of cuproptosis (Left). Abnormal death of intestinal epithelial cells can lead to the occurrence of ulcerative colitis, but the cuproptosis driver genes were identifified as down-regulated in UC patients (Right).

UC is characterized by recurring inflammatory episodes limited to the colon’s mucosal layer (6). Promoting mucosal healing, even at the cell level, has been a potential strategy for IBD, including UC, treatment (7). Therefore, inhibiting abnormal intestinal epithelial cell death to maintain intestinal mucosal homeostasis is a potential intervention strategy. Both studies validated their results using DSS-induced colitis mouse models. Yang et al. conducted a quantitative analysis of four cuproptosis genes using western blot, which further validated the results from their bioinformatics analysis; however, whether the samples used in the experiment were whole colon tissue or intestinal mucosal cells has not been mentioned. The use of intestinal mucosal cells alone for validation will provide more accurate results, as the bioinformatics analysis samples were also from the intestinal mucosa of patients with IBD.

More studies are still needed to verify whether cuproptosis occurs in the intestinal mucosal cells of patients with IBD. Additionally, the detection of cell copper is also necessary.

## Author contributions

All authors listed have made a substantial, direct, and intellectual contribution to the work and approved it for publication.
